# Tragacanth Gum/Chitosan Polyelectrolyte Complexes-Based Hydrogels Enriched with Xanthan Gum as Promising Materials for Buccal Application

**DOI:** 10.3390/ma14010086

**Published:** 2020-12-27

**Authors:** Joanna Potaś, Emilia Szymańska, Anna Basa, Anita Hafner, Katarzyna Winnicka

**Affiliations:** 1 Department of Pharmaceutical Technology, Medical University of Białystok, Mickiewicza 2c, 15-222 Białystok, Poland; joanna.potas@umb.edu.pl (J.P.); esz@umb.edu.pl (E.S.); 2 Department of Physical Chemistry, Faculty of Chemistry, University of Białystok, Ciołkowskiego 1K, 15-245 Białystok, Poland; abasa@uwb.edu.pl; 3 Department of Pharmaceutical Technology, University of Zagreb, Domagojeva 2, 10000 Zagreb, Croatia; ahafner@pharma.hr

**Keywords:** tragacanth gum, xanthan gum, chitosan, polyelectrolyte complex, hydrogel, buccal delivery

## Abstract

Polyelectrolyte complexes based on the electrostatic interactions between the polymers mixed are of increasing importance, therefore, the aim of this study was to develop hydrogels composed of anionic tragacanth gum and cationic chitosan with or without the addition of anionic xanthan gum as carriers for buccal drug delivery. Besides the routine quality tests evaluating the hydrogel’s applicability on the buccal mucosa, different methods directed toward the assessment of the interpolymer complexation process (e.g., turbidity or zeta potential analysis, scanning electron microscopy and Fourier-transform infrared spectroscopy) were employed. The addition of xanthan gum resulted in stronger complexation of chitosan that affected the hydrogel’s characteristics. The formation of a more viscous PEC hydrogel with improved mucoadhesiveness and mechanical strength points out the potential of such polymer combination in the development of buccal drug dosage forms.

## 1. Introduction

Among various routes of drug administration, buccal mucosa is recognized as particularly useful for either local or systemic drug delivery. It is a multilayer structure mainly composed of phospholipids with the lining of non-keratinized stratified squamous epithelium and the underlying tissues rich with blood vessels and capillaries [1]. Due to the large surface area (approximately 50 cm^2^), abundant vascularization, low enzymatic activity, and high permeability, it is found to be an attractive alternative route for orally administered drugs. Furthermore, it offers high accessibility for topically administered medications in the therapy of oral cavity diseases, including infections or inflammatory conditions [1]. Nevertheless, despite the advantages mentioned above, barriers of buccal drug delivery, such as constant flowing down of saliva within the oral cavity, or the risk of accidental swallowing of the product administered, are still challenging [1]. To overcome these limitations, buccal mucoadhesive systems with high retentivity are necessary for the efficient delivery of therapeutic agents. For this purpose, highly mucoadhesive, natural, biocompatible and biodegradable polysaccharide materials can be particularly useful. Among oromucosal drug dosage forms, hydrogels are considered as easy to prepare drug carriers specifically suitable for the treatment of oral cavity conditions. A crosslinked polymeric matrix of a hydrogel is found to enable controlled release of the incorporated drugs with a simultaneous protective effect on damaged tissues [1,2,3].

Over the last few years, polyelectrolyte complexes (PECs)—a three-dimensional network prepared by electrostatic interactions between the oppositely charged polymers [4]—have been recognized as a promising platform for buccal drug administration [5,6,7]. There are several anionic polymers able to form PECs, e.g., sodium alginate, pectin, or glycosaminoglycans, whereas, among cationic materials, mainly chitosan (CS) and its derivatives are recognized as useful [6,8,9]. CS is a natural-origin, non-toxic, biocompatible, and biodegradable polysaccharide [10,11,12], consisted of β (1,4)-linked D-glucosamine and N-acetyl-d-glucosamine units [13]. Because of its multifunctional character, CS has attracted much attention as a compound of mucosal formulations [14,15,16]. Besides high mucoadhesiveness [17], it exhibits antimicrobial [18], anti-fungal [19], anti-inflammatory, and hemostatic properties [20]. Factors limiting the applicability of CS are pH-dependent solubility and high susceptibility to ions resulting in poor mechanical and rheological characteristics and uncontrolled swelling. Combining CS with other polymers is the strategy to bypass these limitations [21].

Among different materials used for the formation of PECs with CS dedicated for mucosal administration, polyanionic natural gums with anionic carboxylic acid groups, such as gellan [22], Arabic [23] and xanthan gum (XG) [24], arouse great interest of researchers, while the combination of CS with tragacanth gum (TG) is a relatively new technological approach.

TG is a natural plant gum being a mixture of different monosaccharides, mainly D-galacturonic acid, derived from *Astragalus species* [25]. Aqueous dispersions of TG are composed of an anionic, water-soluble fraction of tragacanthin or tragacanthic acid (20–30%) and the insoluble-but-swellable-in-water bassorin (60–70%). High stability over a wide range of pH and temperature, emulsifying and viscosity enhancement properties, or biocompatibility determine its widespread use in the food, cosmetic, and pharmaceutical industry [26].

XG is an anionic polysaccharide composed of D-glucose, D-mannose, and D-glucuronic acid units, produced by *Xanthomonas campestris* [27,28]. It is commonly used as a viscosity enhancer, stabilizing, and prolonged-release agent improving the applicability of other materials used in pharmaceutical technology [29,30]. Due to high mucoadhesiveness, its potential in the development of mucosal systems [31] has been evaluated. Similar to TG, XG is regarded as highly stable over a wide range of pH and temperature [29].

Because of the high potential of buccal mucosa in both local and systemic drug delivery, the development of novel buccal mucoadhesive platforms is worth interest. We aimed to create the semisolid PEC-based carriers since numerous clinical data still point out the need to develop mucoadhesive and biocompatible buccal systems for well-known as well as new therapeutic agents, which may eliminate the problem of low retentivity at the site of application and then improve their topical effect. Existing data indicate the usefulness of chitosan PECs in the technology of buccal drug dosage forms with antifungal azoles (e.g., miconazole [7], fluconazole [32]) used in the oral candidiasis treatment, antibiotics (e.g., tetracycline hydrochloride [33]) particularly useful in the prevention and therapy of periodontitis or anesthetic agents (e.g., mebeverine hydrochloride [34]) dedicated for dental procedures. Among buccally administered systemic drugs, analgesic systems with tramadol hydrochloride were also investigated [5].

PEC-based hydrogels composed of TG and CS with or without XG as potential drug carriers were designed. The improved strength and mucoadhesiveness of the hydrogels were expected due to XG incorporation. Furthermore, XG aimed to enhance electrostatic interactions with CS and therefore increase the probability of the stable tripolymeric complex formation. Besides the routine quality tests, including the mechanical properties, pH, or rheological analyses, gels with different ratios of polyanionic gums to CS were analyzed with regard to their mucoadhesive strength and swelling ability. In addition, to have better insight into the character of polymer–polymer interactions, turbidity test and zeta potential measurements for liquid di- and tripolymeric dispersions, as well as Fourier-transform infrared spectroscopy (FTIR) and scanning electron microscopy (SEM) for the freeze-dried PECs, were employed.

## 2. Materials and Methods

### 2.1. Materials

Low-molecular-weight CS (from snow crabs (*Chionoecetes opilio*), shrimps or squids; the degree of deacetylation: 79.9%, determined by the titration method [35]; viscosity: 31–70 mPa·s at 20 °C for 1% CS in 1% acetic acid; average molecular weight: 232 kDa, measured by Agilent 1260 Infinity GPC/SEC at 35 °C with a refractive index detector (Agilent Technologies, Santa Clara, CA, USA) and PSS Novema column (PSS Standards Polymer Service GmbH, Mainz, Germany)) was obtained from Heppe Medical CS GmbH (Haale, Germany). TG (from *Astragalus gummifer*, composed of different polysaccharides, e.g., tragacanthin and bassorin; average molecular weight: 840 kDa [29]) and XG (from *Xanthomonas campestris*, composed of a β (1–4)-d-glucopyranose glucan backbone with side chains of (1–3)-α-d-mannopyranose-(2–1)-β-d-glucuronic acid-(4–1)-β-d-mannopyranose on alternating residues) were purchased from Sigma-Aldrich (St. Louis, MO, USA; average molecular weight: 1000 kDa [29]). Disodium hydrogen phosphate, potassium dihydrogen phosphate, sodium acetate, and propylene glycol were from Chempur (Piekary Śląskie, Poland). Dental hydrogel with 0.2% chlorhexidine digluconate (Elugel, Pierre Fabre, France, series G00526, expiry date 11.2022). The 80% acetic acid was from Avantor Performance Materials Poland S.A. (Gliwice, Poland). Porcine buccal mucosa was received from the veterinary service of the local slaughterhouse (Turość Kościelna, Poland) and stored at −20 °C.

### 2.2. Preparation of the Hydrogels

5% (*w/w*) TG dispersion (pH 4.72) was prepared by the gradual addition of the polymer to the mixture of propylene glycol (5%, *w/w*) and water at room temperature by using a magnetic stirrer. After polymer moistening, mixing was continued at 400 rpm for at least 1 h. For the formulations with XG (pH 4.67), XG at the concentration of 0.5% (*w/w*) was added to TG dispersion at the stage of initial mixing and then stirred at 400 rpm as above. 4% (*w/w*) solution of CS (pH 4.80) was prepared by dissolving polymer in 1% (*v/v*) acetic acid at 40 °C. Formulations were obtained by simple mixing of the individual polymers solutions by using mortar and pestle. Depending on the target ratio of the polyanions to CS, an accurately weighed amount of acetic acid solution of CS was gradually added to TG (F1–F3) or TG/XG mixture (F4–F6) (Table 1). TG and CS at the weight ratio of 7:1 did not create a homogenous matrix, and this formulation was excluded from further experiments.

### 2.3. pH Analysis

pH measurements were performed by using a glass electrode of the pH-meter Orion 3 Star (Thermo Scientific, Waltham, MA, USA) at 25 °C ± 1 °C.

### 2.4. Evaluation of Mechanical Properties

The analysis was performed using TA.XT. Plus texture analyzer (Stable Micro Systems, Godalming, UK) equipped with a 5 kg load cell and backward extrusion measuring system A/Be. For this purpose, a Plexiglas disc with 25 mm-diameter was pushed into 20 g of the hydrogel with a speed of 2 mm/s to a depth of 3 mm. The test parameters were selected according to the study by Tai et al. [36]. Hardness, consistency, and adhesiveness were determined by Texture Exponent 32 software (Stable Micro Systems, Godalming, UK).

### 2.5. Viscosity Measurement

Measurements were performed using viscometer IKA^®^ ROTAVISC me-vi (IKA^®^—Werke GmbH and Co. KG, Staufen, Germany) at 25 °C ± 2 °C at 10,000 rpm, at a torque value ranged from 20% to 30%. Viscosity values were noted after 1 min of the test.

### 2.6. Rheological Properties Analysis

The analysis was performed at 37 °C ± 0.5 °C by using Brookfield viscometer (RVDV-III Ultra, Brookfield Engineering Laboratories, Middleboro, MA, USA) equipped with CPA52Z cone. Due to the immeasurable viscosity of highly viscous hydrogels under the set test parameters (shear rate 2–12 s^−1^, shearing time 30 s), all measurements were performed after dilution of the formulations with simulated saliva (SS) at the ratio of 1:1 (*w/w*) according to the expected saliva absorption at the site of product administration. SS composed of 0.1 M disodium hydrogen phosphate and 0.1 M potassium dihydrogen phosphate with pH adjusted to 6.8 by sodium hydroxide was used [37].

### 2.7. Evaluation of Mucoadhesiveness by Gravimetric Test on an Inclined Temperature-Controlled Plane

**Figure 1:**
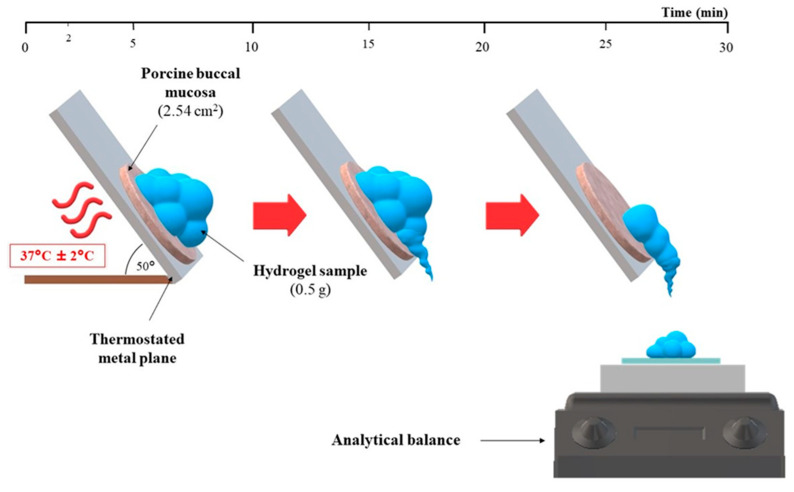
The scheme of the gravimetric test on an inclined temperature-controlled plane.

Mucoadhesive properties of hydrogels were assessed by using a self-constructed apparatus consisting of the thermostated metal surface and the analytical balance (Figure 1), according to recommendations of Sandri et al. with modifications [38,39]. 0.5 g of each hydrogel was placed on a defrosted porcine buccal mucosa (2.54 cm^2^ discs) at 37 °C ± 2 °C. After 3 min of thermostatting, the metal surface with the sample was set at the angle of 50°, and sample behavior was observed for the next 30 min. At the set time intervals (2, 5, 10, 15, 20, and 30 min), the mass of hydrogel which flowed down was noted. In order to provide the highest correlation of the in vitro/in vivo conditions, the carriers were mixed with SS at 1:0.25 and 1:0.5 weight ratios based on salivary flow rate [40]. Following the above procedure, the retentivity of the commercially available product was also assessed.

### 2.8. Swelling Study

**Figure 2:**
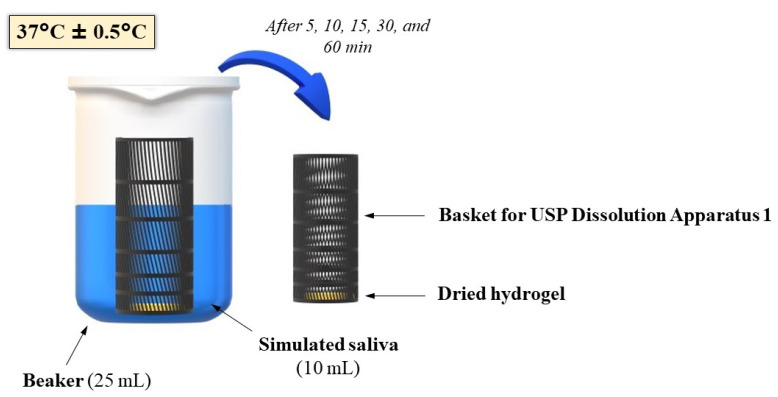
The scheme of the swelling test.

A swelling study was performed for dried hydrogels. 700 mg ± 5 mg of each formulation was placed in a plastic blister packaging with a diameter of 13 mm and dried in the oven at 40 °C for 24 h. Accurately weighted films were located in baskets for USP Dissolution Apparatus 1 [41] and immersed in 10 mL of SS at 37 °C ± 0.5 °C. At the set time intervals, baskets with films were carefully drained and weighted (Figure 2). For evaluation of the swelling capacity, the degree of swelling (α) was determined as follows:

α (%) = (Ws − Wo)/Wo × 100,(1)

where Ws—weight of hydrogels after swelling, Wo—initial weight of dried hydrogels [42].

### 2.9. Turbidity Measurement

Turbidity measurement was performed using a Hach Model 2100 N IS^®^ Laboratory Turbidimeter. All results were presented in a nephelometric turbidity unit (NTU), which signifies the amount of scattered light reaching the detector. Comparing to the studied hydrogels (Table 1), dispersions with a fifty-fold lower concentration of the polymers were prepared; however, the qualitative and quantitative composition of the dispersing media for both CS and TG/XG has remained unchanged. The accurately weighed amount of CS solution was added dropwise to 30 g of TG or TG/XG dispersion under stirring. Different weight ratios of TG to CS (XG to CS) were used: 30:1 (3:1), 20:1 (2:1), 15:1 (1.5:1), 10:1 (1:1), 7:1 (0.7:1), 5:1 (0.5:1). The formulation with the precipitated PECs was then homogenized at 6000 rpm for 1 min in order to unify the particle size and transferred it to the sample cell. All results were recorded within 3 min of PEC formation [43].

### 2.10. Zeta Potential and Conductivity Measurement

Zeta potential and conductivity of the PECs precipitates were determined with Zetasizer NanoZS90 (Malvern Instruments, Malvern, UK). For this purpose, the polymers mixtures with the weight ratio of TG to CS (XG to CS) 20:1 (2:1), 10:1 (1:1), and 7:1 (0.7:1), corresponding to the composition of the studied hydrogels, were tested.

### 2.11. SEM Analysis

5 g of the representative dispersions with precipitated di- and tripolymeric complexes with TG/CS ratio of 10:1 and 7:1 were placed in glass vials after separation from the majority of the polyelectrolyte-depleted solution phase and subjected to 12 h freeze-drying process at −20 °C by using freeze-dryer Christ Alpha 1–2 LDplus (Donserv, Warsaw, Poland) equipped with rotary vane pump RZ 2.5 (Vacuubrand, Wertheim, Germany). The lyophilizates were evaluated with scanning electron microscopy (Inspect™S50, FEI Company, Hillsboro, OR, USA). Samples were placed on adhesive tapes fixed to the surface of a special stand and gold sprayed.

### 2.12. FTIR Analysis

The chemical structure of the freeze-dried PECs was determined by the attenuated total reflection FTIR spectroscopy (ATR-FTIR) (NicoletTM 6700, Thermo Scientific, Madison, WI, USA, equipped with a DTGS detector and diamond ATR-crystal). The spectra were recorded in the range of 500–4000 cm^−1^ at a resolution of 4 cm^−1^.

## 3. Results and Discussion

### 3.1. Preparation and Physicochemical Characteristics of the Hydrogels

There were no significant differences in the pH values between the formulations based on TG and the CS and those with XG addition regardless of CS concentration (Table 2). Although the pH of the hydrogels (about 5.0) was lower than the physiological pH of saliva (5.5–7.0 [40]), it cannot be treated as the factor limiting the applicability of formulated carriers. Comparable pH values eliminated potential discrepancies in the hydrogels characterization resulting from different solubility of the polymers and, therefore, different amounts of dissociated functional groups available for electrostatic interactions. In fact, among various factors, which affect PECs formation, pH as close as possible to pKa of individual polymers determines the high-efficiency of the interpolymer complexation [24].

Due to the noticeable differences in the appearance and the application properties of the gels, analysis of mechanical properties, including hardness, consistency, and adhesiveness, was found to be a useful tool for better insight into their structures. While hardness is defined as a force required to attain a change in the structure of a sample, the consistency parameter describes the work of a probe necessary for this deformation. Adhesiveness allows in turn to quantify the work necessary to break down the interaction between a probe and an analyzed sample. With adding of a less viscous CS solution, an evident reduction in mechanical strength was observed; however, a significantly higher drop of hardness, consistency, and adhesiveness was recorded for dipolymeric formulations. The incorporation of XG improved the mechanical strength of the hydrogels and resulted in homogenous tripolymeric systems formation. Analogically to the visible, more fluid character of F2 and F6 gels, the preparations performed the lowest values of the mechanical parameters, and F2 was noted as the weakest semisolid platform (Table 2). Low values of hardness and consistency testified to the low resistance of the samples to mechanical stress applied inter alia at the stage of withdrawing from a container or spreading at the application site.

**Figure 3:**
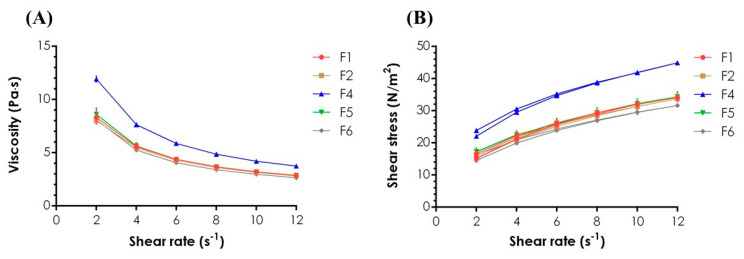
Plots of viscosity vs. shear rate (**A**) and rheograms (**B**) for the hydrogels diluted with simulated saliva (SS) at the weight ratio of 1:1 determined at 37 °C ± 0.5 °C (mean ± SD, *n* = 3).

Viscosity measurements performed for undiluted hydrogels confirmed the observations made in the texture analysis, and no substantial difference between F1 and F4 viscosity values was noted. Mixing the oppositely charged polymers most probably resulted in the formation of a system consisting of PEC coacervates and polyelectrolytes-depleted polymeric matrix with correspondingly lower viscosity and mechanical strength [4]. Rheological analyses performed for the formulations after dilution with SS at 37 °C ± 0.5 °C pointed in turn out the significant influence of ions and temperature on the PECs viscosity (Figure 3A,B). While for XG/CS hydrogels developed by Martínez-Ruvalcaba et al. [44], the addition of Na^+^ resulted in more viscous system preparation, observations made by Mohammadifar et al. [45] on TG fractions derived from *A. gossypinus* pointed out the significant viscosity reduction after the dispersion of the polymer in ionic media. High-temperature tends to lower XG [46] and TG [45] viscosities; however, conformational changes in XG chains at above 36 °C lead to a more anionic character of the polymer [46] and then might be a reason for more intense PECs formation. Increasing CS concentration resulted in turn in gradual complexation of the polyions [47] and then the formation of a stoichiometric PEC with completely neutralized functional groups what led to the viscosity stabilization despite the polyanion/polycation ratio. The plots of viscosity against shear rate (Figure 3A) pointed out the non-Newtonian pseudoplastic formulations. All gels exhibited shear-thinning behavior—upon the growth of shear rate, the viscosity decreased [48]. According to the rheograms (Figure 3B), the formulations were characterized by low thixotropic properties as evidenced by minor hysteresis loops formed between the ascending and descending curves.

### 3.2. Mucoadhesion Properties

Development of each drug dosage form for mucosal application should cover the assessment of its mucoadhesion properties. The ability of a product to adhere to a mucus layer is key for prolonged drug delivery at the site of administration. Buccal drug delivery is particularly challenging when it comes to the selection of highly mucoadhesive polymers resistant to a continuous process of flowing down of saliva within the oral cavity. Three types of testing methods for the evaluation of mucoadhesion, including physical, mechanical, and dynamic indirect tests, can be distinguished [38]. To maintain a high correlation of the in vitro/in vivo conditions, we decided to measure the retention time of the hydrogels by using a gravimetric test on an inclined plane. This is an example of a dynamic indirect test in which thermostated buccal porcine mucosa was used as a model tissue, and gels were diluted with SS to mimic the physiological clearance mechanism from the mucosal site.

**Figure 4:**
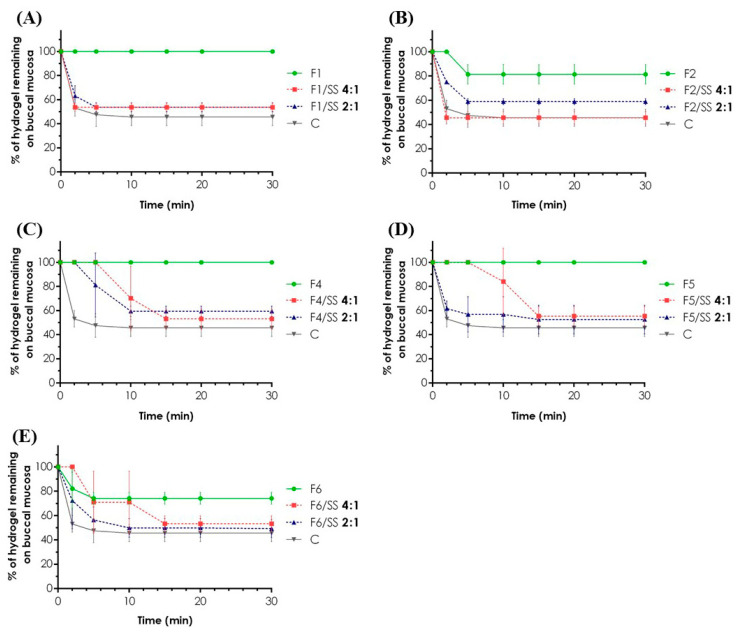
In vitro mucoadhesive properties of the carriers determined as% of a hydrogel/diluted hydrogel F1 (**A**), F2 (**B**), F4 (**C**), F5 (**D**), and F6 (**E**) remaining on the porcine buccal mucosa over 30 min of an experiment at 37 °C ± 2 °C. Commercially available product (C—control) was used in its undiluted form as a reference (mean ± SD, *n* = 3).

Tripolymeric complexes revealed noticeably higher retentivity than formulations without XG (Figure 4A–E), and the polyanion addition probably resulted in the mucoadhesive properties improvement. For all tested carriers, the amount of gel remaining on the mucosa ranged from 69 to 100%. While for F4 and F5 formulations, no loss of an applied sample over 30 min was recorded, a two-fold increase in CS concentration in F2 resulted in 19% ± 8% reduction in the retentivity in the first 5 min of the measurement. After 2 min, the depletion of the F6 sample of more than 20% was also observed.

Dilution of F4–F6 hydrogels with SS resulted in the retention of 53% ± 8% of the applied sample after 15 min (Figure 4C–E). The results pointed out the low retentivity of the formulations in the first 15 min of the experiment. While for undiluted F1 and F2 gels, a difference in mucoadhesion properties was easy to note, no significant discrepancies were observed for the corresponding samples mixed with SS. Interestingly, despite a two-fold increase in the SS content in F1/SS 2:1 and F2/SS 2:1, almost the same behavior of the gels on the model tissue was recorded. Regardless of the degree of the sample dilution, approximately 50% of each hydrogel was maintained on the porcine buccal mucosa over 30 min observation.

Due to the low mechanical properties of the commercially available product and then numerous difficulties over its maintenance on the model tissue during a thermostatting process, the measurements were limited to the undiluted samples of the hydrogel. Except for F2/SS 4:1 (Figure 4B), the retentivity of the product was, on average, lower than the developed hydrogels and their mixture with simulated saliva. After 30 min test, 46% ± 7% of the applied product remained on the buccal mucosa. The obtained results are very promising in terms of the observations made for designed formulations since they highlight significantly higher values of retentivity as compared to the gel used as a reference.

### 3.3. Swelling Ability

The swelling behavior of a buccal hydrogel reflects on a further drug release profile. In fact, a swellable polymeric matrix may be responsible for the prolonged release of a drug incorporated [49]. Furthermore, it affects such elements of a gel performance as mucoadhesiveness, enhancement of the drug absorption by opening the mucosal tight junctions [50], or a tolerance of a product at the site of application due to a volume increase. Transport of water from a mucus layer to a swellable polymeric matrix leads to gradual dilution of a sample what may influence its residence time, but on the other side, it increases the intensity of mucus cohesion, being one of the mechanisms of the mucoadhesive joints formation [51].

**Figure 5:**
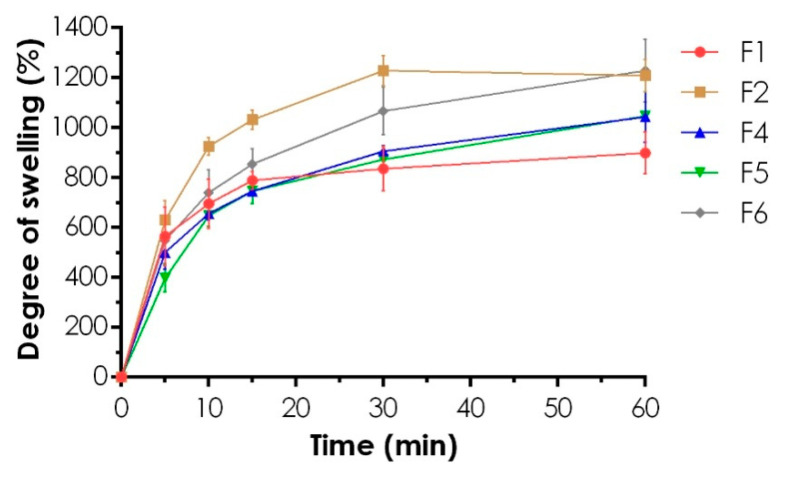
The swelling profiles of the hydrogels in SS expressed as the degree of swelling (mean ± SD, *n* = 3).

The swelling test revealed significantly higher values of the degree of swelling by 400% for F2 formulation compared to F1, as shown in Figure 5. Flattening the curves, which was noted especially for F2 after 60 min, resulted from a loss of a highly diluted sample at the stage of drying of a basket. In contrast to XG and CS with the proven water-uptake ability which slows down the polymer matrices disintegration [52], TG is subjected to erosion upon contact with water, most probably due to its chemical structure—rapidly dissolving tragacanthin/tragacanthic acid and hydrophobic bassorin with a limited swelling capacity [53]. Comparing to the very similar kinetics of swelling recorded for F4 and F5 gels, for F6, a substantial increase of saliva uptake was noted. Since any similarities in gels swelling behavior were highly correlated with the mechanical and viscosity characteristics recorded for undiluted formulations, weaker and less viscous PECs matrices (F2, F6) may be more susceptible for SS penetration and then swelling. The presence of the ionized amino groups results in their repulsion with simultaneous relaxation of the CS matrix highly susceptible to medium uptake [54]. The negligible difference in swellability of the tripolymeric formulations F4 and F5 was most probably due to the complete neutralization of CS amino groups by carboxylic acid groups of gums. However, the further increase of CS content in F6 resulted in the preparation of a non-stoichiometric PEC with an excess of free CS able to swell what was confirmed in zeta potential measurement for fifty-fold lower polymers concentrations (see Section 3.5. below). For none of the gels tested, the state of swelling equilibrium was recorded within 60 min. While the shortest rehydration time was noted for F2 (below 15 min), only F1 did not achieve the mass before the drying process within 60 min. As mentioned above, tripolymeric hydrogels maintained their initial shape forming a highly swollen matrix, whereas, in dipolymeric formulations, progressive disintegration of the membranes resulting in unwanted leakage of a sample from a basket was observed.

### 3.4. Turbidity

**Figure 6:**
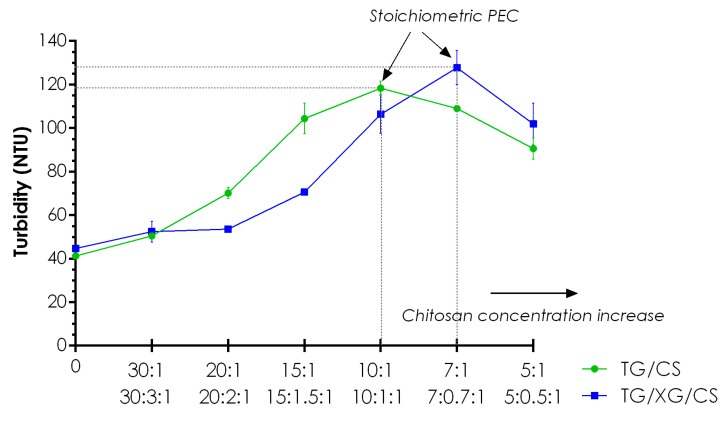
The measured turbidity values of tragacanth gum (TG)/chitosan (CS) and TG/xanthan gum (XG)/CS formulations with regard to various polyanion/polycation ratios.

The aim of the test was to confirm the process of PECs formation between cationic CS and anionic gums under experimental conditions. Fifty-fold lower concentrations of the polymers compared to the studied hydrogels enabled visualization of the primary PECs formation by measuring the turbidity of the dispersions obtained. Di-and tripolymeric complexes revealed a similar tendency of turbidity changes upon CS concentration increase (Figure 6); however, the addition of XG affected the formation of noticeably greater particles. Furthermore, for the tripolymeric complexes, characteristic displacement of the plot toward higher CS concentrations was observed what may be explained by the different ability of light scattering reduced by the effect of light absorption by tripolymeric precipitates. The polymer ratios for which the highest turbidity values were recorded may be considered as the points of stoichiometric PEC formation in which all negatively charged carboxylic acid groups of gums became electrostatically bonded to positively charged amino groups of CS what resulted in precipitation of the largest amount of PEC [43]. For dipolymeric mixtures, the highest turbidity was noted for the sample with a ten-fold higher concentration of TG than CS, while for tripolymeric formulations, TG/XG/CS 7:0.7:1 showed the maximum turbidity. Partial correlation of the results for tripolymeric complexes with zeta potential values presented in Section 3.5. may arise from the most intense electrostatic interaction between TG and CS at the missed ratio between 10:1 and 7:1. After the point of stoichiometric PEC formation, excess of charged CS probably determined the repulsion of cationic amino groups of the polymer leading to PEC destabilization with the weaker ability of light scattering.

### 3.5. Zeta Potential and Conductivity

Zeta potential measurements pointed out the significant differences in the ionic strength of the polyanions used (Table 3). For dipolymeric mixtures with a ratio of 20:1, dominance of the negatively charged particles of TG with, on average, the zeta potential of −13.9 mV was observed. The presence of free cationic CS resulted in the formation of the second peak with a mean value of 28.7 mV. The almost neutral charge of TG/CS 10:1 particles may be a reason for maximum interaction between the polymers what is consistent with nephelometric analysis. The positive charge of TG/CS 7:1 probably corresponded to the presence of a neutralized dipolymeric core surrounded by a slight excess of cationic CS. The addition of XG has affected the electrokinetic behavior of the formulations and resulted in significantly lower values of zeta potential for non-stoichiometric tripolymeric complexes with TG/CS ratios of 20:1 and 10:1. According to the results obtained, no point of complete neutralization of gums carboxylic acid groups by amino groups of CS can be indicated. The positive charge of the formulation with the ratio of 7:1 points out the non-stoichiometric PEC formation with the excess of positive CS. While upon increasing of CS concentration in dipolymeric dispersions, a gradual increase of conductivity was indicated, which may have arisen from progressive depletion of free polyelectrolytes in the solution during the process of PEC precipitation, for TG/XG/CS 7:0.7:1 significant decline of the parameter was recorded. Bobreshova et al. [55] have shown a minor conductivity of low-molecular weight CS, comparing to typical polyanions, so the significant excess of free CS at the ratio of 7:0.7:1 may hamper the charge transfer what results in the conductivity drop.

### 3.6. Morphology of the PECs

**Figure 7:**
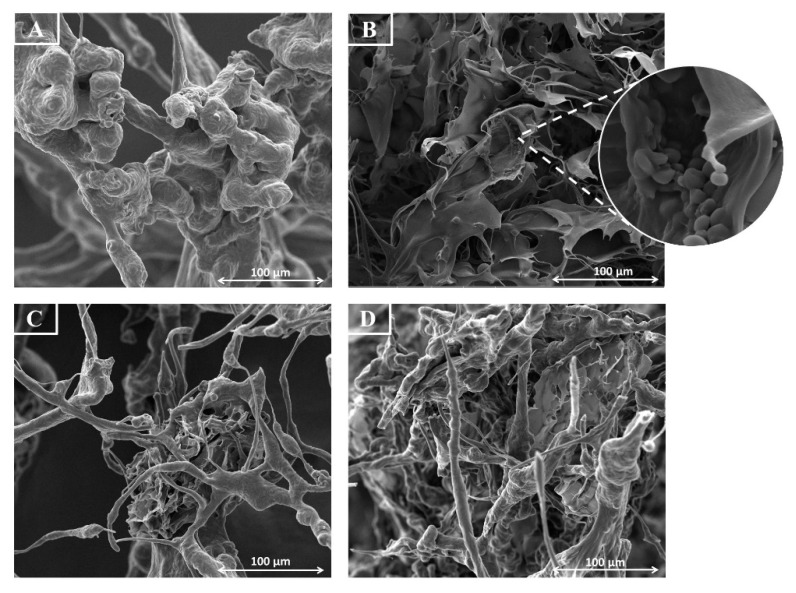
Representative SEM images for dipolymeric systems with TG/CS ratio of 10:1 (**A**) and 7:1 (**B**), and tripolymeric formulations with TG/XG/CS ratio of 10:1:1 (**C**) and 7:0.7:1 (**D**) under 1000× magnification (inset—under 10,000×).

SEM analysis distinguished two morphologically different PECs systems. Freeze-drying of the dispersions resulted in the formation of the sponge-like structure with aggregates of PECs particles shaped in the homogenization process. Comparing to the relatively homogenous dipolymeric matrix (Figure 7A,B), the addition of XG affected a binary character of the lyophilizate—thin “filaments” of TG/CS PECs were surrounded by the highly branched structure of XG/CS and/or TG/XG/CS PECs (Figure 7C,D).

### 3.7. FTIR Analysis

**Figure 8:**
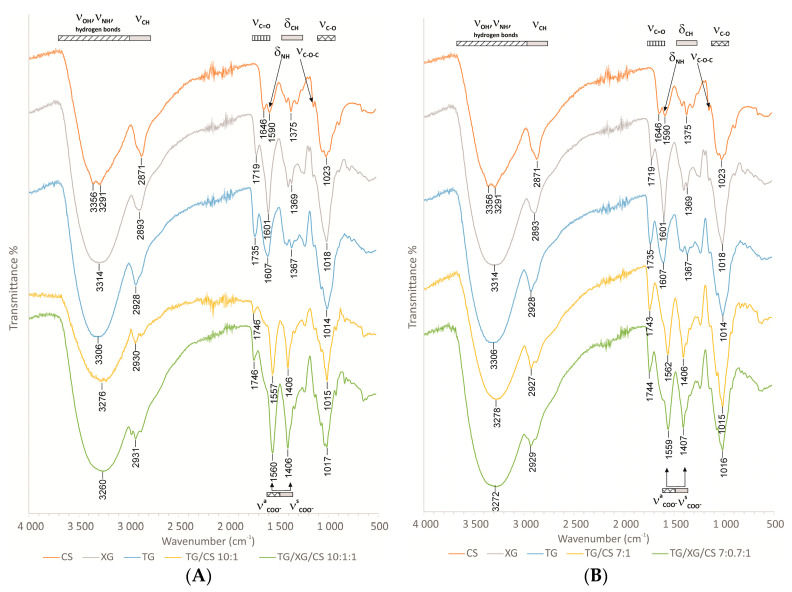
FTIR spectra of CS, TG, XG, di- (TG/CS 10:1 and TG/CS 7:1) and tripolymeric complexes (TG/XG/CS 10:1:1 and TG/XG/CS 7:1:1) (**A**,**B**) with different polymer ratios.

Comparing to the FTIR spectra of the single polymers, the analysis of the di- and tripolymeric complexes pointed out the multiple hydrogen bonding due to the shifts observed for OH (proton donor) and C=O (proton acceptor) groups stretching in the range of 3400–3200 cm^−1^ and 1680–1650 cm^−1^, respectively (Figure 8A,B). New bands, absent in spectra of single polymers, related to asymmetric COO^−^ stretching (1610–1550 cm^−1^) and symmetric COO^−^ stretching (1420–1300 cm^−1^) was observed for di- and tripolymeric complexes. The formation of carboxylic acid groups able to ionic interactions with amino groups of CS may be confirmed. Due to the relatively low polycation/polyanion ratios, deformation vibrations of NH_3_^+^ may be hardly visible and stretching, which was expected at the wavelength of 3380–3280 cm^−1^, was overlapped with the OH stretching mentioned above. Asymmetric bending vibrations of NH_3_^+^ observed at 1600 cm^−1^ overlap with stretching vibrations of C=O shifted to lower wavenumbers because of hydrogen bonding. Additionally, for water molecules adsorbed in the samples, the overlap band at 1646 cm^−1^ was observed. Symmetric bending vibrations of NH_3_^+^ at 1300 cm^−1^ overlapped with bending vibrations of OH and CH bonds [56,57,58,59,60]. Complicated IR spectra with many overlapping bands of various bonds make a more detailed analysis impossible.

## 4. Conclusions

Optimization of the ratio of the polymer is a key factor for successful PEC-based hydrogels preparation. While for di- and tripolymeric hydrogels with the lowest polyanion/polycation ratio, only negligible physicochemical variations were recorded, escalating complexation of XG by increasing amount of CS significantly affected the formulations mechanical and rheological properties. The incorporation of XG resulted in the formation of a more viscous PEC hydrogel with improved mucoadhesiveness and mechanical strength, particularly advantageous for buccal drug dosage forms. It should be mentioned that despite the satisfactory behavior of placebo hydrogels on the buccal mucosa, incorporation of drugs with different charges or solubility may affect the polymer-polymer interactions and then the interpolymer complexation process. According to this, further studies directed toward the implementation of buccally administered active substances are in progress and will be discussed in due course.

## Figures and Tables

**Table 1:** Composition of the hydrogels.

Formulation	Polymers Ratio (*w/w*)	
	TG:CS ^1^	XG:CS ^1^
F1	20:1	–
F2	10:1	–
F3	7:1	–
F4	20:1	2:1
F5	10:1	1:1
F6	7:1	0.7:1

^1^ TG—tragacanth gum, XG—xanthan gum, CS—chitosan.

**Table 2:** pH and mechanical properties of the hydrogels (mean ± SD, n = 3).

Formulation	pH	Mechanical Properties			Viscosity (Pa·s)
		Hardness (g)	Consistency (g·s)	Adhesiveness (g·s)	
F1	5	191 ± 13	250 ± 6	250 ± 26	92 ± 0
F2	5	24 ± 0	31 ± 0	27 ± 1	19 ± 0
F3	not determined ^1^				
F4	5	210 ± 18	258 ± 11	272 ± 30	91 ± 0
F5	5	107 ± 3	114 ± 1	100 ± 13	50 ± 2
F6	5	34 ± 1	39 ± 1	40 ± 6	19 ± 1

^1^ The formulation was excluded from further analyses due to the observed phase separation.

**Table 3:** Zeta potential and conductivity of the di- and tripolymeric dispersions (mean ± SD, *n* = 3).

Polymers Ratio (*w/w*)		Zeta Potential (mV) [%]	Conductivity (mS/cm)
TG:CS ^1^	XG:CS ^1^		
20:1	–	Peak 1: −13.9 ± 5.7 [66.6 ± 14.1] Peak 2: 28.7 ± 5.8 [33.4 ± 14.1]	0.48 ± 0.01
10:1	–	0.2 ± 0.3 ^2^	0.72 ± 0.04
7:1	–	3.2 ± 0.1 ^2^	0.97 ± 0.03
20:1	2:1	–34.4 ± 12.4 ^2^	0.50 ± 0.03
10:1	1:1	–21.3 ± 2.6 ^2^	0.82 ± 0.02
7:1	0.7:1	27.2 ± 3.3 ^2^	0.27 ± 0.01

^1^ TG—tragacanth gum, XG—xanthan gum, CS—chitosan. ^2^ For these samples, only one peak was recorded.

## Data Availability

Detailed data are available on request from the corresponding author.
